# Miscellaneous

**Published:** 1889-12

**Authors:** 


### MISCELLANEOUS.

### YOUR BILLIONS OF ANCESTORS.

Did you ever think how many male and female ancestors were required to bring you into the world ? First it was necessary that you should have a father and mother—that makes two human beings. Each of them must have had a father and mother—that takes four more human beings. Again each of them must have had a father and mother—making eight more human beings. So on we go back to the time of Jesus Christ—fifty-six generations. The calculation thus resulting shows that 139,235,017,489,534,976 births must have taken place in order to bring you into this world ! You, who read these lines. All this since the birth of Christ. Not since the beginning of time. According to Proctor, if from a single pair, for 5,000 years, each husband and wife had married at twenty-one years of age, and there had been no deaths, the population of the earth would be 2,199,915, followed by 144 ciphers. It would require to hold this population a number of worlds, the size of this, equal to 3,166,526, followed by 125 ciphers. The human mind shrinks in contemplating such immense numbers.—*St. Louis Republic.*

### THE NEXT CENTURY.

There is only one century that I would rather be an inhabitant of than the present one, and that is the next. I do not care to live in any one that is past, but I would like to see the next one. I would like to see how some of these movements that are going on will come out,—what will be the changes in the social, the religious, the political life ; what the next step in discovery, in conquest, of this wonderful earth of ours will be. And, if the end is not eternal silence, I expect to know. I expect to keep the run of these movements, even if I go to some distant planet. If I am engaged in work that will take me to a distance, I will get the news, or I will come back again now and then and see for myself. If that theory is true, just think of it for a moment ! How would you enjoy seeing gathered in some great hall to-day the company of all the immortals that have distinguished the history of our race by their physical, their intellectual, their moral, or their spiritual glories ? How would you like to look upon the face of Shakespeare, to see if Dante had got rid of that sadness that he wore, to talk with Gœthe, to hear the music of Mozart and Mendelssohn ? If this theory is true, we shall meet all these ; we shall find them and so have in our grasp all the past of the earth and watch the growth of all the future. No wonder that Socrates' mind kindled at the thought, and he said, "If this be so, then let me die again and again," if this be the condition.

Rev. M. J. Savage.

---

The Doctor's Pay.—Medical men in general are probably not aware that in France, at least, the doctor's claim on the estate of a diseased patient has precedence of all others. Even the landlord's claim for arrears of rent must yield to the doctor's fee, The courts of Rouen, Poictiers, and the Seine have alike decided that as it is an imperative right of humanity that the dying should have the necessary care and treatment, such attendance should be paid for before all the other debts.

